# Retraction Note to: LATS2 overexpression attenuates the therapeutic resistance of liver cancer HepG2 cells to sorafenib‑mediated death via inhibiting the AMPK–Mfn2 signaling pathway

**DOI:** 10.1186/s12935-019-1084-7

**Published:** 2019-12-30

**Authors:** Jie Song, Wei Zhao, Chang Lu, Xue Shao

**Affiliations:** 1 Department of Hepatopancreatobiliary Medicine, The Second Hospital of Jilin University, Changchun, 130000 China; 2 Department of Pharmacy, The Second Hospital of Jilin University, Changchun, 130000 China; 3 Department of Anesthesiology, The Second Hospital of Jilin University, Changchun, 130000 China

## Retraction to: Cancer Cell Int (2019) 19:60 10.1186/s12935-019-0778-1

The editor has retracted this article [1] because Fig. 2f has been duplicated from Fig. 6j in a previously published article [2]. In addition, the article contains sections that substantially overlap with a previously published article [3]. The data reported in this article are therefore unreliable.

Xue Shao agrees to this retraction. Jie Song, Wei Zhao, and Chang Lu have not responded to any correspondence from the editor/publisher about this retraction.
